# Supplementary material for: Fine mapping of the antigenic epitopes of the Gc protein of Guertu virus
Source: PLoS One. 2022 Jul 26;17(7):e0271878. doi: 10.1371/journal.pone.0271878 (PMC9321374; doi:10.1371/journal.pone.0271878)
Supplement: S1 Raw images — (PDF) [file pone.0271878.s001.pdf]

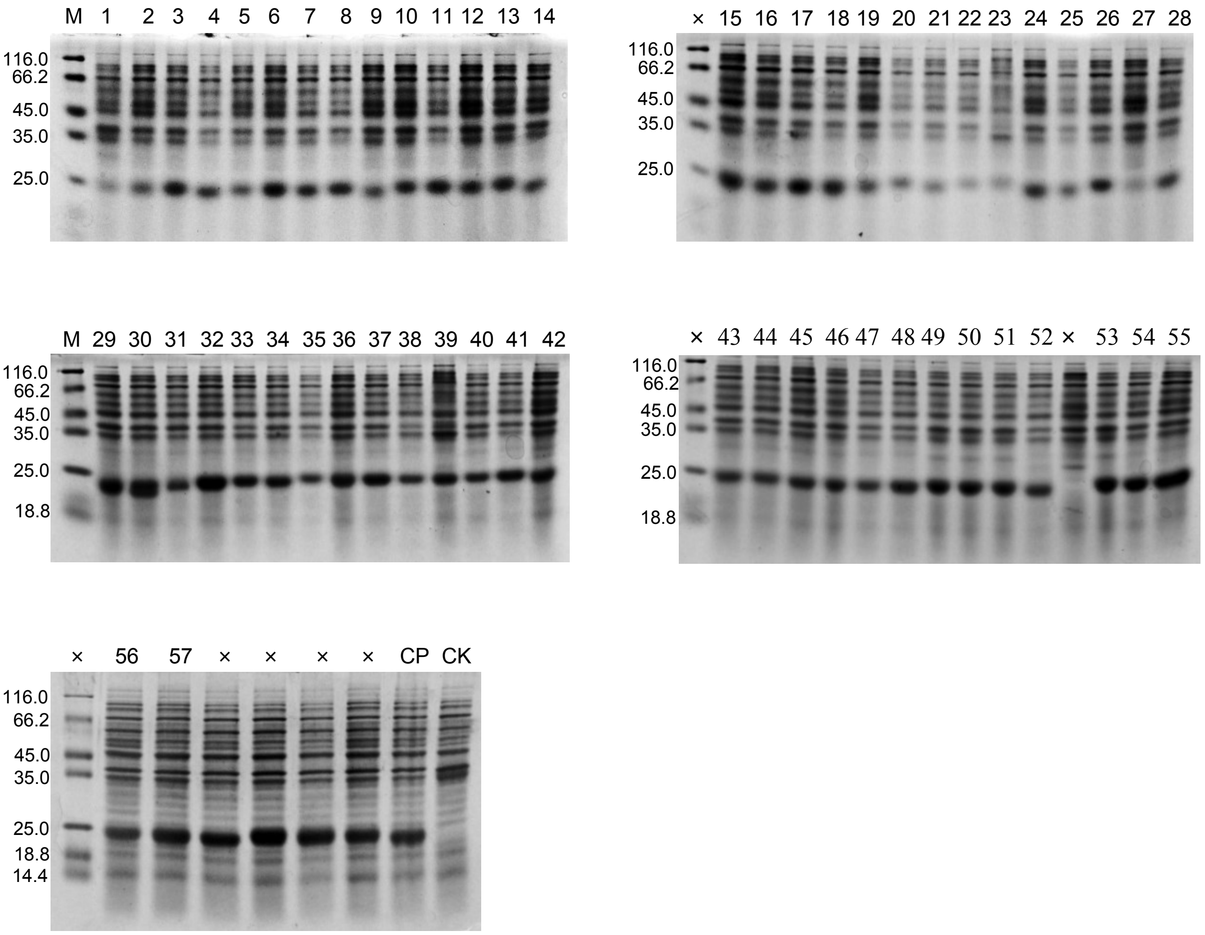

Fig 2. SDS-PAGE and Western blot analysis of expressed 16mer-peptides. (A and C) SDS-PAGE analysis of expressed 16mer-peptides. The numbers of P1-P57 indicate each 16mer-peptide in cell total proteins. The cell proteins of each r-clone were resolved by 12% SDS-PAGE gel electrophoresis and stained with Coomassie brilliant blue.M, the protein molecular marker; CP, Positive control of expressed 16mer peptide in GTV-NP; CK, Negative control of GST188 carrier protein expressed by pXXGST-2.

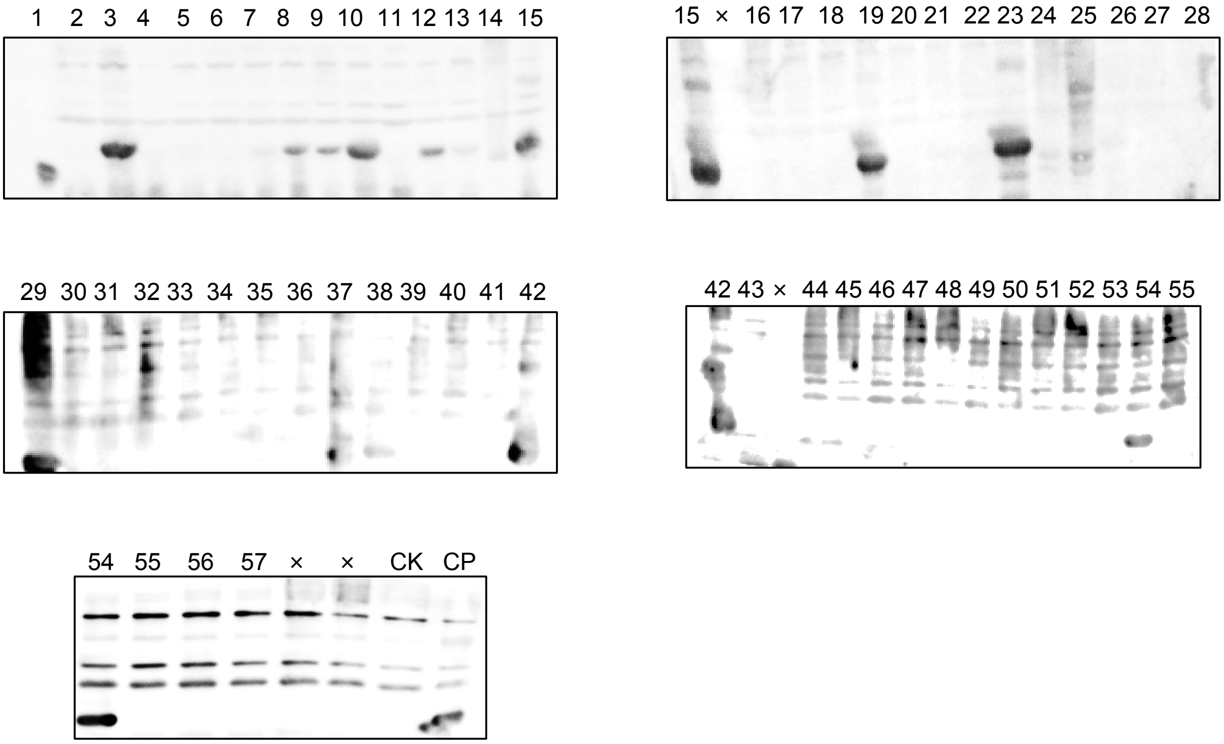

Fig 2. SDS-PAGE and Western blot analysis of expressed 16mer-peptides. (B and D) Western blot analysis for mapping reactive 16mer peptides in P1-P57. The rabbit antiserum (1:300 dilution) against GTV-Gc was used in Western blotting. The reactive bands in Western blotting were visualized by enhanced chemiluminescence.

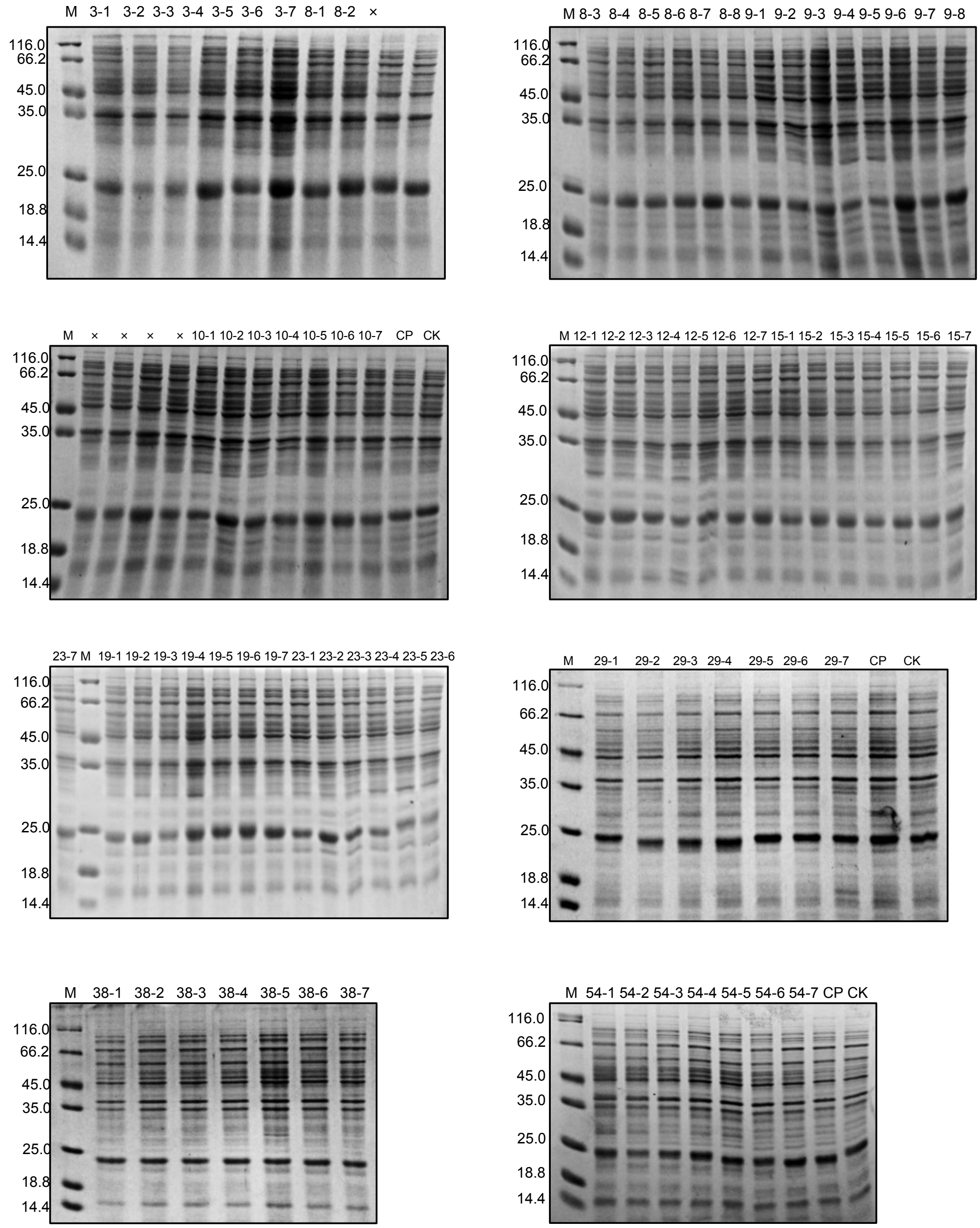

Fig 3. SDS-PAGE and Western blot analysis of expressed 8mer-peptides. (A, C, E and G) SDS-PAGE analysis of expressed 8mer-peptides. It indicates each 8mer peptide of P3, P8, P9, P10, P12, P15, P19, P23, P29, P38 and P54. The cell proteins of each r-clone were resolved by 12% SDS-PAGE gel electrophoresis and stained with Coomassie brilliant blue. M, the protein molecular marker; CK, Negative control of GST188 protein. CP, Positive control of mapped reactive P3.

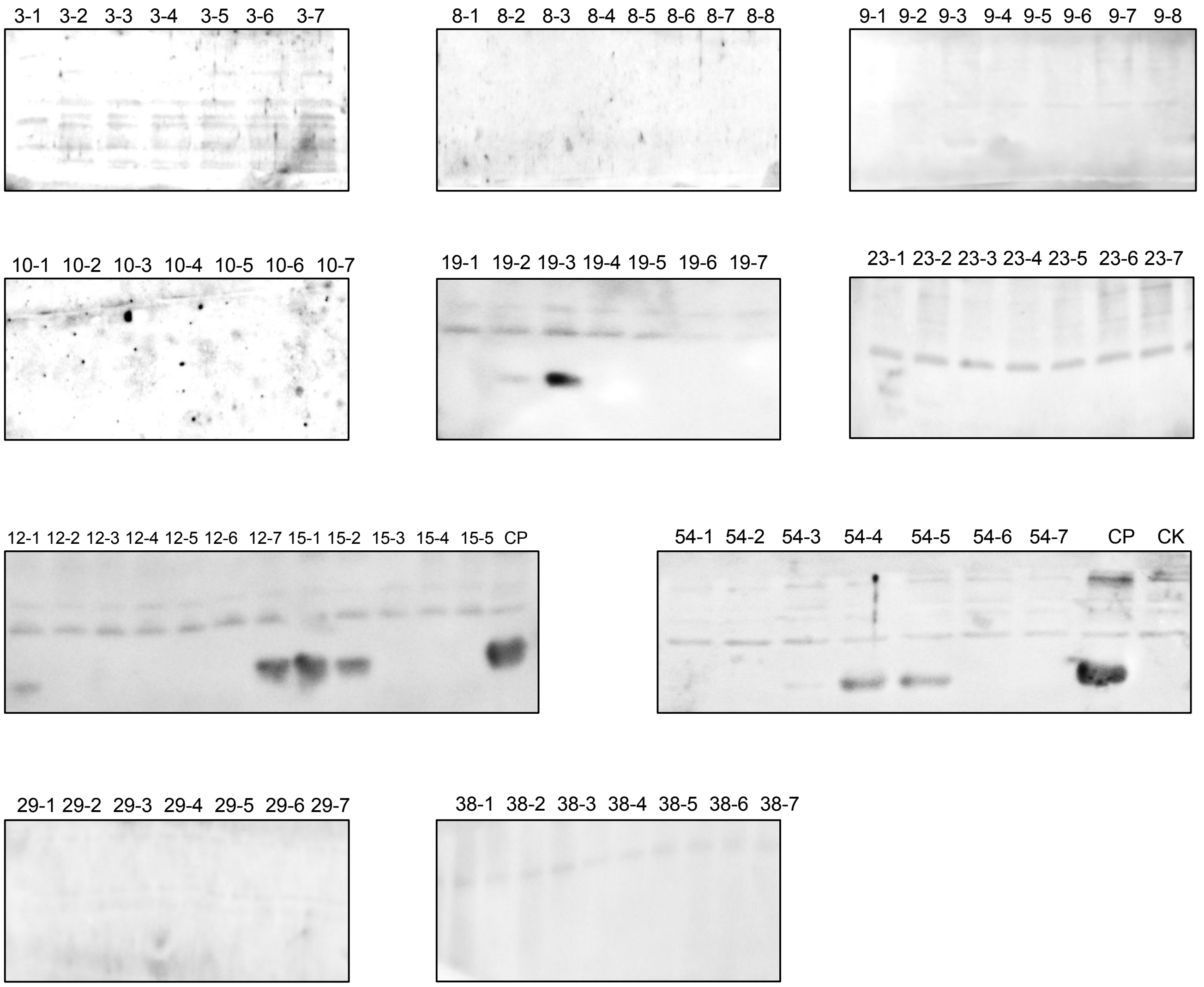

Fig 3. SDS-PAGE and Western blot analysis of expressed 8mer-peptides. (B, D, F and H) Western blot analysis for mapping fine epitopes in each reactive 16mer-peptides. The rabbit antiserum against GTV-Gc (1:300 dilution) was used in Western blotting. The reactive bands in Western blotting were visualized by enhanced chemiluminescence.

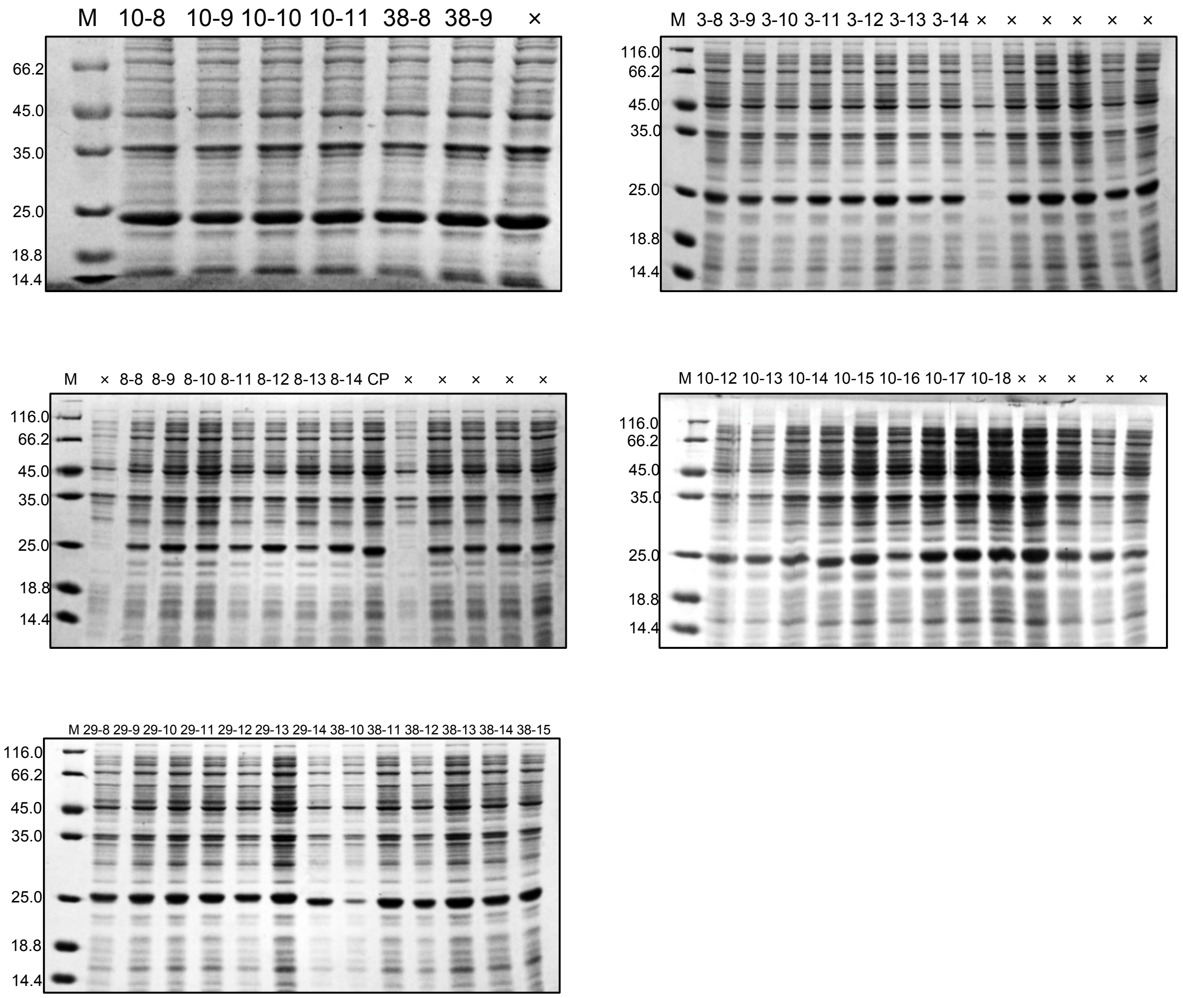

Fig 4. SDS-PAGE and Western blot analysis of expressed 10mer peptides. (A and C) SDS-PAGE analysis of expressed10mer-peptides. It indicates each short peptide for numbers 10mer of P3, P8, P10, P29 and P38. The cell proteins of each r-clone were resolved by 12% SDS-PAGE gel electrophoresis and stained with Coomassie brilliant blue. M, the protein molecular marker; CK, Negative control of GST188 carrier protein. CP, Positive control of P3.

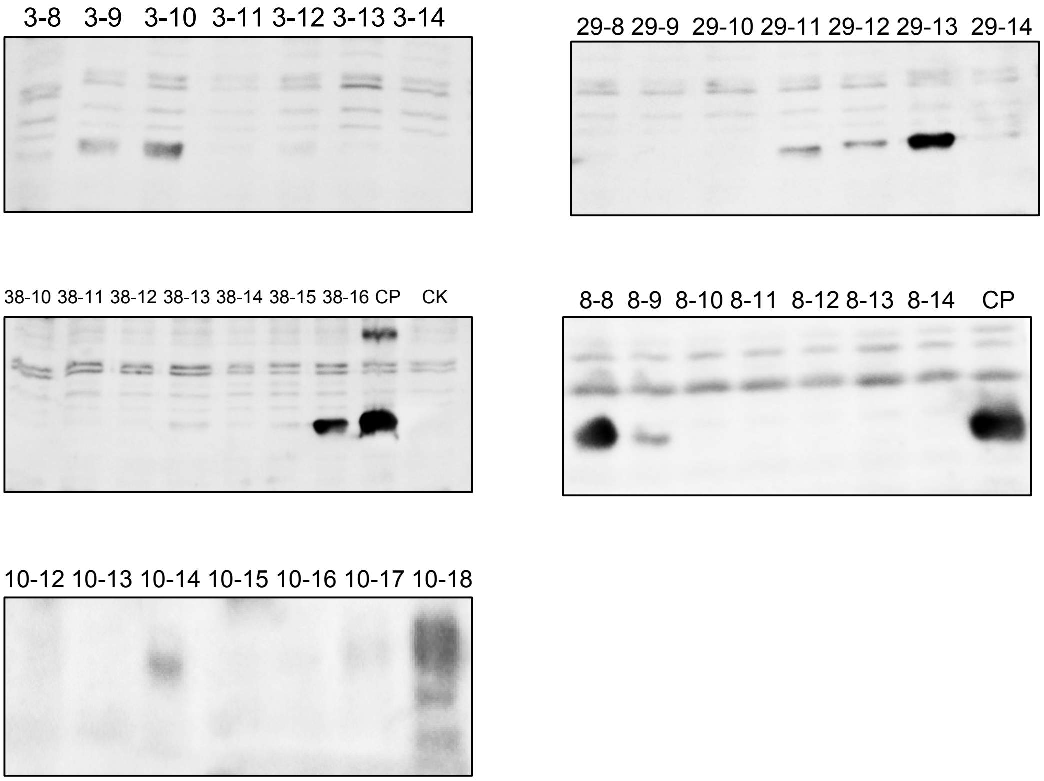

Fig 4. SDS-PAGE and Western blot analysis of expressed 10mer peptides. (B and D) Western blot analysis for mapping fine epitopes in each reactive 10mer-peptide. The rabbit antiserum against GTV-Gc (1:300 dilution) was used in Western blotting. The reactive bands in Western blotting were visualized by enhanced chemiluminescence.

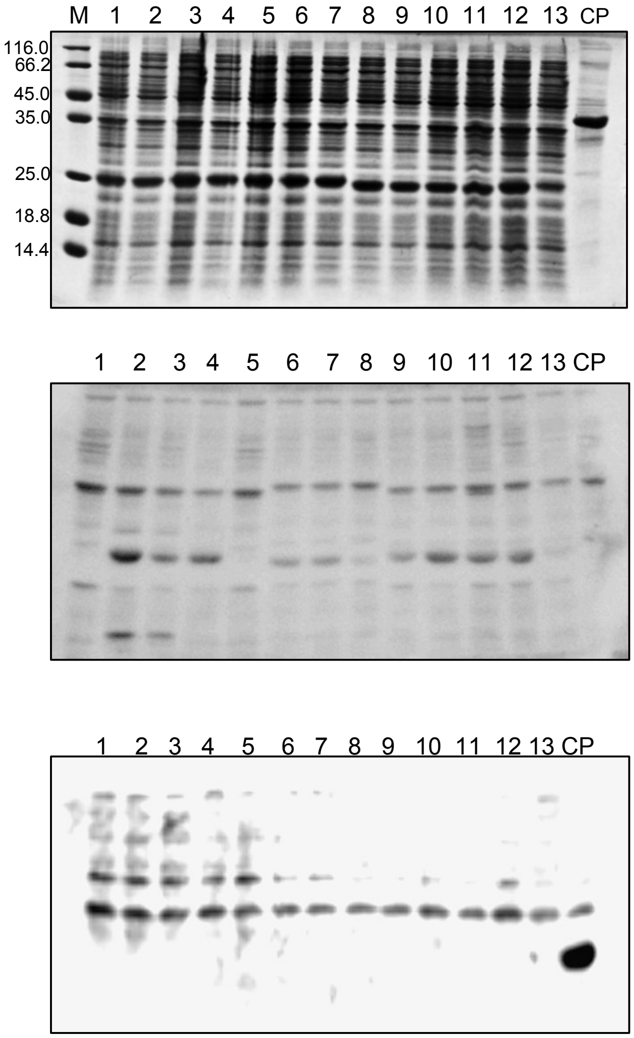

Fig 6. Western blot analysis of mapped BCEs using sheep sera. (A) SDS-PAGE analysis of expressedBCEs. (B) Using a positive serum from a sheep confirmed GTV-infection. (C) Using a serum from healthy sheep with no history of GTV infection as a negative control. NC, Negative control of GST188 protein. PC, Positive control of GTV-Gc1.
