# Supplementary material for: Fine mapping of the antigenic epitopes of the Gc protein of Guertu virus
Source: PLoS One. 2022 Jul 26;17(7):e0271878. doi: 10.1371/journal.pone.0271878 (PMC9321374; doi:10.1371/journal.pone.0271878)
Supplement: Attachment — Submitted filename: Response to Reviewers.docx [file pone.0271878.s002.docx]

Response to Reviewers

Dear Academic Editor Zheng Xing:

Thank you very much for your letter and advice. We have revised the manuscript entitled “Fine mapping of the antigenic epitopes of the Gc protein of Guertu virus”, and we would like to re-submit it for your consideration. We also appreciate all the critical comments from you and reviewers. With these improvements, we hope that the current version can meet the Journal’s standards for publication. The following is a point-by-point response to all those comments and a list of changes we have made to the manuscript. We have addressed the comments raised by the reviewers, and the amendments are highlighted in red in the revised manuscript. Point by point responses to the reviewers’ comments are listed below in this letter, and the responses are highlighted in blue.

We would like to express our sincere thanks to the reviewers for the constructive and positive comments.

I look forward to hearing from you soon.

With best wishes,

Yours sincerely,

Surong Sun, Prof.

Xinjiang Key Laboratory of Biological Resources and Genetic Engineering, College of Life Science and Technology, Xinjiang University, Urumqi 830046, PR China

E-mail: sr_sun2005@163.com Tel/Fax: 086-991-8582554

1. In your point-by-point response to reviewer’s comments, you need to indicate whether you’ve revised your manuscript accordingly, and how and where.
   Expression for lack of epitope response in sheep serum: Amino acid sequences of B-cell epitopes are not universal among species. Epitopes need to be processed by proteases and recognized by the MHC complex for presentation to the surface of cell membrane in antigen presenting cells. Differences in protease processing and MHC presentation of antigen epitopes (either B or T cell epitope) exist naturally among different species of animals. Your explanation does not make much sense (Lines 279-81).

**Response:** Sorry, a paragraph in lines 279-281 is not accurate enough and has been deleted. The specific amendments are as follows: the Western blot results showed that there was a good consistency between the immune response of rabbit and sheep to GTV GC protein.

2. Figure 7 & 8 are missing. Somehow we can’t find them.

**Response:** Thanks for the good remind. Figure 7 & 8 have been attached to files.

3. Carefully edit the manuscript to remove errors in spelling, expression, and gramma.

**Response:** Thanks for the suggestion. We have removed all errors in the Manuscript. For example, "fifine" revised as "fine" in line 77, "immunedominant" revised as " immunodominant" in line 266, and so on.

Minor Issues:

1. There are many errors with spelling, expression, or English gramma. These errors need to be corrected. Please carefully edit the manuscript before resubmission. For example:

Line 66: “… which is most closely related to GTV 66 evolution”

**Response:** Thanks, we have rewritten “which is most closely related to GTV 66 evolution” as “At present, the virus cell receptor binding domain of SFTSV is located on glycoprotein Gc” in line 65 in the revised manuscript.

Line 77: “… are as no…”

**Response:** Thanks, we have rewritten this sentence in line 77 of the revised manuscript.

Line 144: “…the GTV Gc protein was designed as fifty-seven 16-mer peptides…”

**Response:** Thanks, we have rewritten “The GTV Gc protein was truncated into fifty-seven 16mer-peptides using modifified overlapping peptide biosynthesis” as “The GTV Gc protein was designed into fifty-seven 16mer-peptides using modified overlapping peptide biosynthesis” in line 143 of the revised manuscript.

Line 208: 16mer-peptide or 16mer peptide, with or without hyphen? Be consistent in the text and all figure legends. Please check.

**Response:** Thanks, we have changed all “16/10/8mer peptide” as “16/10/8mer-peptide” in the manuscript to with hyphen.

Line 209: SDS-PAGE gel electrophoresis: GE in PAGE represents gel electrophoresis, No need to repeat. Correct this in all figure legends. Please check.

**Response:** Thanks, we have deleted gel electrophoresis, and revised all the “SDS-PAGE gel electrophoresis” as “SDS-PAGE” in the manuscript.

Line 210: blue.M : You need a space between the two words (letters). There are several other places with similar spelling errors in the text or figure legends. Please check.

**Response:** Spaces have been added and the whole manuscript been checked and corrected. For example, " blue.M " revised as " blue. M " in line 210, " bufferedsaline " revised as " buffered saline " in line 180, and so on.

Line 212: Western blot (212), Western blotting (213, 214), western blot analysis (221), or western blotting analysis (269)? Be consistent in the text and all figure legends. Please check.

**Response:** Thanks, all Western blot (212), Western blotting (213, 214), western blot analysis (221), or western blotting analysis (269) in the manuscript has been unified as “Western blot analysis”.

Line 209: What is r-clone?

**Response:** Thanks for the good reminding, “r-clone” revised as “recombinant clone” in lines 207, 234, and 258 in the revised manuscript.

Line 253: EGc11 and EGc12 should not have the same aa numbers from 230 to 237.

**Response:** Thanks, “EGc11 (^230^QAGMGVVG^237^), and EGc12 (^230^RSHDSQGKIS^237^)” revised as “EGc11 (^230^QAGMGVVG^237^), and EGc12 (^303^RSHDSQGKIS^312^)” in line 251 in the revised manuscript.

Line 259: expressed10mer-peptides

**Response:** Thanks, “expressed10mer peptides” revised as “expressed 10mer-peptides” with hyphen on line 257 in the revised manuscript.

Line 259: “It indicates each short peptide for numbers 10mer…” Correct expression?

**Response:** Thanks, we have rewritten “SDS-PAGE analysis of expressed 10mer peptides. It indicates each 10mer peptide for numbesr P3, P8, P10, P29 and P38” as “SDS-PAGE analysis of expressed 10mer-peptides for P3, P8, P10, P29 and P38” in line 257 of the revised manuscript.

2. Page numbers must be inserted on each page for submitted manuscripts.

**Response:** We have been inserted on each page for submitted manuscripts.
