# Supplementary material for: Fine mapping of the antigenic epitopes of the Gc protein of Guertu virus
Source: PLoS One. 2022 Jul 26;17(7):e0271878. doi: 10.1371/journal.pone.0271878 (PMC9321374; doi:10.1371/journal.pone.0271878)
Supplement: Attachment — Submitted filename: Response to reviewers.docx [file pone.0271878.s003.docx]

Response to Reviewers

Dear Academic Editor Tetsuro Ikegami:

Thank you very much for your letter and advice. We have revised the manuscript entitled “Fine mapping of the antigenic epitopes of the Gc protein of Guertu virus”, and we would like to re-submit it for your consideration. We also appreciate all the critical comments from you and reviewers. With these improvements, we hope that the current version can meet the Journal’s standards for publication. The following is a point-by-point response to all those comments and a list of changes we have made to the manuscript. We have addressed the comments raised by the reviewers, and the amendments are highlighted in red in the revised manuscript. Point by point responses to the reviewers’ comments are listed below in this letter, and the responses are highlighted in blue.

We would like to express our sincere thanks to the reviewers for the constructive and positive comments.

I look forward to hearing from you soon.

With best wishes,

Yours sincerely,

Surong Sun, Prof.

Xinjiang Key Laboratory of Biological Resources and Genetic Engineering, College of Life Science and Technology, Xinjiang University, Urumqi 830046, PR China

E-mail: sr_sun2005@163.com Tel/Fax: 086-991-8582554

1. In Discussion, authors should clearly describe the limitations of presented study, which identified only linear epitopes of Gc proteins. It should include (1) current study does not predict B cell epitopes affected by different MHC haplotypes, (2) the mechanism of virus neutralization via Gc proteins remains unknown, and (3) the neutralizing role via identified linear epitopes of Gc proteins has not been characterized.

**Response:** Thanks for the good remind. In the Discussion section we describe in detail the limitations of this study, and described in lines 394-397 as “Among study limitations, this study only identified linear epitopes of the Gc protein. Also, there are currently no B cell epitopes predicted to be affected by different MHC haplotypes. The mechanism of virus neutralization by anti-Gc protein antibodies remains unknown, and neutralization through identified linear epitopes of Gc protein has not been characterized.”

2. Line 21 to 22: Gc proteins are shielded by Gn protein in virions. Gn protein is responsible to receptor binding, while Gc protein serves as a fusion protein upon entry with low pH in endosomes. Although Gc proteins encode neutralizing epitope, authors should describe the role of Gc accurately with appropriate citations.

**Response:** Thanks for the good remind. We have added corresponding references in lines 21-25.

3. Line 47: “tick bite-transmitted” should be “tick-borne”.

**Response:** Thanks for the suggestion. We have revised “tick bite-transmitted” as "tick-borne" in line 47.

4. Line 49: “Research studies on..” should be “Studies on..”

**Response:** Thanks, we have rewritten “Research studies on” as “Studies on” in line 49 in the revised manuscript.

5. Line 55: “The researchers” should be “Researchers”

**Response:** Thanks, we have rewritten “The researchers” as “Researchers” in line 55 in the revised manuscript.

6. Line 60–61: Gn and Gc proteins are considered to be cleaved co-translationally, but not post-translationally.

**Response:** Thanks, we have revised “M segment that encodes a glycoprotein precursor that is cleaved into two mature envelope proteins (Gn and Gc) during  post-translationally modification” as “M segment that encodes a glycoprotein precursor that is cleaved into two mature envelope proteins (Gn and Gc) during co-translational modification” in line 60–61 of the revised manuscript.

7. Line 65: “At present, the virus cell receptor binding domain of SFTSV is located on glycoprotein Gc [11].” This description is not supported by cited reference.

**Response:** Thanks, we have added other related referencs in the revised manuscript.

8. Line 78 – 98: This paragraph describes the method for this study, which is a standard strategy to test overlapped peptides as fusion recombinants with GST. Therefore, this paragraph should be removed from introduction section. Line 100: “using the improved BSP strategy and rabbit anti-GTV-Gc pAbs” should be written as “using biosynthetic peptide (BSP) methods (Ref) and rabbit anti-GTV-Gc pAbs”.

**Response:** Thanks for the good suggestion, we have removed the description “The BSP method had the following features: (1) By selecting the truncated GST188 protein as the fusion tag for the recombinant expression of short peptides, the fragment size of the expressed fusion peptide was maintained within 21.5-22.5 kDa, which allowed its easy identification by sodium dodecyl sulfate–polyacrylamide gel electrophoresis (SDS-PAGE) and subsequent western blot analysis. This also facilitated the easy distinction of the short peptides from the proteins expressed by the host (Escherichia coli); (2) The antigenic proteins were first divided into 16mer-peptides with eight overlapping amino acid residues, following which basic and complete screening was performed for those containing BCEs; (3) Further truncation of the positive 16mer-peptides into 8mer-peptides with seven overlapping amino acid residues allowed the precise determination of the minimal epitope motif for each linear BCE [15].”, and put it in the methods section under “Strategy for designing GTV Gc overlapping peptides”. And “using the improved BSP strategy and rabbit anti-GTV-Gc pAbs” rewritten as “using biosynthetic peptide (BSP) methods and rabbit anti-GTV-Gc pAbs” in lines 80–90 in the manuscript.

9. Line 80: “The BSP method developed by Xu et al. in 2009 was created for the…” should be “The BSP method developed by Xu et al. in 2009 was used for the…”.

**Response:** Tanks, we revised as " The BSP method developed by Xu et al. in 2009 was created for the…” as “The BSP method developed by Xu et al. in 2009 was used for the” in line 80.

10. Line 81–82: “the research group led by Xu Wanxiang successfully improved upon their method to conduct fine mapping..” should be “the same research group successfully mapped linear…”.

**Response:** Thanks, we revised “the research group led by Xu Wanxiang successfully improved upon their method to conduct fine mapping..” as “the same research group successfully mapped linear…”in line 81-82.

11. Line 111 – 113: “The animal serum samples were collected using a random sampling method that did not involve sacrifice of the animals [15].” This description should clarify the following point: “serum collection was performed according to this approved animal protocol.”

**Response:** Thanks for the good reminding, we added “serum collection was performed according to this approved animal protocol.” in “Ethical statement” section in line 103 in the revised manuscript.

12. Line 114: “Vectors, strains and antibodies” section requires to be separated: “Plasmids”, and “Antibodies”. PDVF membrane or ECL explanations should be moved to western blot section. “Plasmids” section should combine “Construction of the truncated Gc fragments for recombinant plasmid expression (line 129 – 137).

**Response:** Thanks, the “Vectors, strains and antibodies” section was separated as “Antibodies” and other method section, “Plasmids” section was combined with “Construction of the truncated Gc fragments for recombinant plasmid expression” (line 113-116) in the revised manuscript.

13. Line 259: Line 116: “GenBank accession no. ALQ33264.1” is for protein sequence. It should be GenBank accession no. KT328592.1 (same with line 131).

**Response:** Thanks, we revised the GenBank accession no as “KT328592.1” line 114 in the revised manuscript.

14. Line 119: Authors should define “Gc1 and Gc2” at their first occurrence in text.

**Response:** Thanks, we have defined “Gc1 and Gc2” as “Gc1 (aa 1-291) and Gc2 (aa 252-549) ” in line 105 of the revised manuscript.

15. Line 120-121: “Taq DNA polymerase, DNA marker, and T4 ligase (TaKaRa Co., Ltd, Dalian, China)”. This partial sentence should be corrected.

**Response:** We have corrected the description in the methods section in manuscripts.

16. Line 120: “License number” may be removed from the text.

**Response:** We have removed from the manuscripts.

17. Line 152-167: “Construction” of plasmid should be combined to “Plasmid” section.

**Response:** We have constructed “Plasmid” with “Construction of the truncated Gc fragments for recombinant plasmid expression” in lines 113-116 in revised manuscript.

18. Line 166: “Heat induction at 45°C was then conducted for another 4 h.” This description requires a citation to justify the protein induction via pET plasmid in BL21 by changing incubation temperature to 45℃, rather than using standard induction using IPTG.

**Response:** We have revised the induction temperature “45℃” as “42℃” in lines 160 and 194 in revised manuscript. However, thermal induction was for pXXGST-3 plasmid, not pET plasmid.We have added the corresponding reference in line 161.

19. Line 173-174:“The strains expressing the correct protein size were provided to Shanghai Sangon for sequencing and strain preservation.” Authors should clarify the meaning of sentence: e.g., strains? Strain preservation?

**Response:** We have revised “The strains expressing the correct protein size were provided to Shanghai Sangon for sequencing and strain preservation” as “The plasmids expressing the correct protein size were send to Shanghai Sangon for sequencing” in lines 167-168 in revised manuscript.

20. Line 216 and 243: “To identify the minimal antigenic epitopes” should be written as “To identify the minimal linear antigenic epitopes”.

**Response:** We have revised “To identify the minimal antigenic epitopes” as “To identify the minimal linear antigenic epitopes” in line 211 in revised manuscript.

21. Line 237: Although text indicated that this analysis shows 8-mer peptides, whereas the legend explains that B, D, F, and H are 16-mer peptides. Authors should provide further clarification.

**Response:** Thanks for the good remind. We have corrected “16-mer peptides” as “8-mer peptides” in line 232 in revised manuscript.

22. Line 313-315: “In future, serological….” This sentence should be removed from Result section.

**Response:** Thanks for the good remind. We have removed the prescription “In the future, serological validation of GTV should be conducted to investigate whether these epitopes are specific to this virus and whether they can be used to detect SFTSV and GTV antibodies or to design broad-spectrum vaccines.” to lines 394-397 in revised manuscript.

23. Line 340: “Bunyaviruses are the largest family of RNA viruses…” should be corrected: e.g., “family Bunyaviridae” no longer exists..

**Response:** Thanks for the good remind. We have revised “Bunyaviruses are the largest family of RNA viruses” as “*Bunyavirales* are the largest order of RNA viruses” in line 333 in revised manuscript.

24. Line 350-351: “could cause murine viremia, organopathological changes, and murine lesions,…” should be “could cause viremia or organopathological changes,…”.

**Response:** Thanks for the good remind. We have revised “could cause murine viremia, organopathological changes, and murine lesions” as “could cause viremia or organopathological changes” in lines 343-344 in revised manuscript.

25. Line 396: Use of monoclonal antibody panels for SFTSV and GTV will likely map the distinct epitopes.

**Response:** Thanks for the good remind. We have revised the conscription in line 396 according to the comments in revised manuscript.
