# Supplementary material for: Fine mapping of the antigenic epitopes of the Gc protein of Guertu virus
Source: PLoS One. 2022 Jul 26;17(7):e0271878. doi: 10.1371/journal.pone.0271878 (PMC9321374; doi:10.1371/journal.pone.0271878)
Supplement: Attachment — Submitted filename: Response to Reviewers.docx [file pone.0271878.s004.docx]

Response to Reviewers

Dear Academic Editor Tetsuro Ikegami:

Thank you very much for your letter and advice. We have revised the manuscript entitled “Fine mapping of the antigenic epitopes of the Gc protein of Guertu virus”, and we would like to re-submit it for your consideration. We also appreciate all the critical comments from you and reviewers. With these improvements, we hope that the current version can meet the Journal’s standards for publication. The following is a point-by-point response to all those comments and a list of changes we have made to the manuscript. We have addressed the comments raised by the reviewers, and the amendments are highlighted in red in the revised manuscript. Point by point responses to the reviewers’ comments are listed below in this letter, and the responses are highlighted in blue.

We would like to express our sincere thanks to the reviewers for the constructive and positive comments.

I look forward to hearing from you soon.

With best wishes,

Yours sincerely,

Surong Sun, Prof.

Xinjiang Key Laboratory of Biological Resources and Genetic Engineering, College of Life Science and Technology, Xinjiang University, Urumqi 830046, PR China

E-mail: sr_sun2005@163.com Tel/Fax: 086-991-8582554

1. Line 22 (Abstract), and line 65 (text): Ref. 10 and 12: No experiments showed that SFTS Gc itself can bind to receptor molecules. Ref 11: Although N-glycosylation sites in Gc protein were described, the functional roles in receptor-mediated entry are speculative. Thus, there is no clear evidence to describe that “virus cell receptor binding domain of SFTSV is located on glycoprotein Gc”.  If authors just describe “N-glycosylation sites is located on SFTSV Gc glycoproteins”, and their hypothetical roles is argued, it might be fine. Glycosylation of proteins plays a role in protein folding and Golgi trafficking, which also supports virus maturation/assembly process.

Response: Thanks for the good remind. Indeed, there is no report proving that there is SFTSV cell receptor binding region on GC. According to your suggestion, we have revised “The viral glycoprotein Gc” as "The viral glycoprotein (GP) " in Line 22 (Abstract), and revised “the virus cell receptor binding domain of SFTSV is located on glycoprotein Gc” as “presumable N-glycosylation sites is located on SFTSV Gc glycoproteins” in line 65 (text).

2. Line 81-82: “More recently, the same research group successfully mapped linear of the linear BCEs of three structural proteins…” needs to be corrected.

**Response:** Thanks for the good remind. We have rewritten “More recently, the same research group successfully mapped linear of the linear BCEs of three structural proteins…” as “and they successfully mapped linear of the linear BCEs of three structural proteins” in line 81.

3. Line 349: Authors should also mention viral fusion, which is a major role of Gc proteins.

**Response:** Thanks for the suggestion. Virtual fusion has been added to the manuscript (line 348).

4. Others: It should be clarified whether N-glycosylation sites of SFSTV Gn and Gc have been demonstrated (requiring citations) or still presumptive. If it has not been shown, all description should be written as “presumable N-glycosylation sites”.

**Response:** Thanks for the suggestion. We have rewritten as “presumable N-glycosylation sites” in line 65.

.
